# Coexistence of Guillain-Barré Syndrome and an Impending Rupture of a Pancreaticoduodenal Artery Aneurysm Associated With Celiac Artery Stenosis

**DOI:** 10.7759/cureus.114756

**Published:** 2026-08-18

**Authors:** Harumasa Shimoda

**Affiliations:** 1 Neurology, Juntendo University, Tokyo, JPN

**Keywords:** celiac artery stenosis, guillain-barré syndrome, hickam’s dictum, pancreaticoduodenal artery aneurysm, visceral artery aneurysms

## Abstract

Guillain-Barré syndrome (GBS) after medical procedures has been reported, although the association is not clear. Since the use of sedatives for the procedures may complicate neurological evaluation and delay the initiation of treatment, all physicians should be familiar with its clinical features. Celiac artery stenosis can increase collateral blood flow through the pancreaticoduodenal arcades and contribute to the formation of arterial aneurysms. The relationship between a pancreaticoduodenal artery aneurysm associated with celiac artery stenosis and GBS is unclear. Multiple life-threatening disorders may coexist by chance; therefore, clinicians should consider an additional underlying condition when a single disorder cannot explain the entire clinical picture.

A 34-year-old woman was transferred to our hospital for the evaluation of progressive muscle weakness in all four limbs and respiratory failure. She had developed flu-like symptoms 10 days before transfer. Two days later, she noticed numbness involving all four extremities and back pain. The following day, her back pain worsened, and she was diagnosed with an impending rupture of a pancreaticoduodenal artery aneurysm associated with celiac artery stenosis. Urgent transcatheter arterial coil embolization resulted in improvement of her back pain, but the numbness persisted. She subsequently reported muscle weakness in all four limbs, which was attributed to the residual effects of sedative medications used during the procedure. The following day, she developed respiratory failure requiring intubation and mechanical ventilation, which prompted her transfer for further management. Based on rapidly progressive muscle weakness with absent reflexes and a positive anti-GM1 antibody test, a diagnosis of GBS was made. Following two courses of intravenous immunoglobulin therapy, she was weaned from mechanical ventilation and eventually was able to walk with a walker.

We learned the following lessons from this case. First, since GBS is difficult to recognize in patients undergoing medical procedures requiring sedative agents, physicians should be familiar with its clinical presentation and maintain a high index of suspicion. Second, as Hickam’s dictum suggests, patients may experience multiple distinct conditions simultaneously. When a single diagnosis cannot explain the entire clinical course, clinicians should consider the possibility of a coexisting pathology.

## Introduction

Guillain-Barré syndrome (GBS) is an acute immune-mediated polyradiculoneuropathy that typically manifests as progressive, symmetric muscle weakness and loss of tendon reflexes [1]. It can rapidly progress to life-threatening respiratory failure [1]. Although the association is unclear, there are cases of GBS presenting after surgeries or medical procedures [2,3]. In those situations, the use of sedative agents may complicate neurological evaluation and delay diagnosis and treatment [4]. Since timely intervention is associated with better outcomes [5], all physicians should be familiar with this condition.

Celiac artery stenosis may reduce blood flow to the foregut [6]. Poor perfusion may be compensated by collateral flow; however, hemodynamic changes contribute to the formation of an aneurysm in small branches such as the pancreaticoduodenal arcades and the dorsal pancreatic artery [7]. This vascular rearrangement may progress silently [8] and may present with acute pain due to the impending rupture of aneurysms [9]. The relationship between GBS and a pancreaticoduodenal artery aneurysm associated with celiac artery stenosis is unclear. Hickam’s dictum is a diagnostic principle, emphasizing that patients may have multiple simultaneous conditions [10]. Although it is extremely rare for two life-threatening conditions to coexist by chance, delayed recognition of either condition may have serious consequences. We, therefore, should not overlook this possibility when clinical scenarios cannot be explained by one pathology.

We report a case of the concomitant occurrence of GBS and an impending rupture of a pancreaticoduodenal artery aneurysm associated with celiac artery stenosis.

## Case presentation

**Figure 1:**
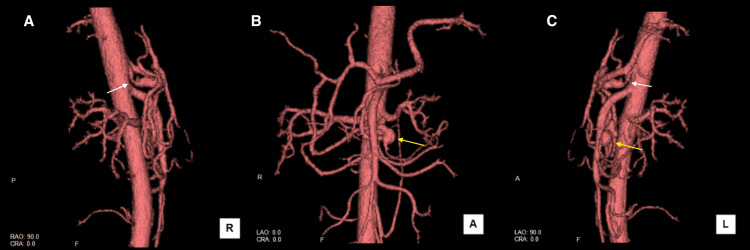
Three-dimensional computed tomography angiography: (A) right lateral view, (B) anterior view, and (C) left lateral view The celiac artery stenosis is indicated by the white arrows on the right lateral view (A) and left lateral view (C). The pancreaticoduodenal artery aneurysm is indicated by the yellow arrows on the anterior view (B) and left lateral view (C).

**Figure 2:**
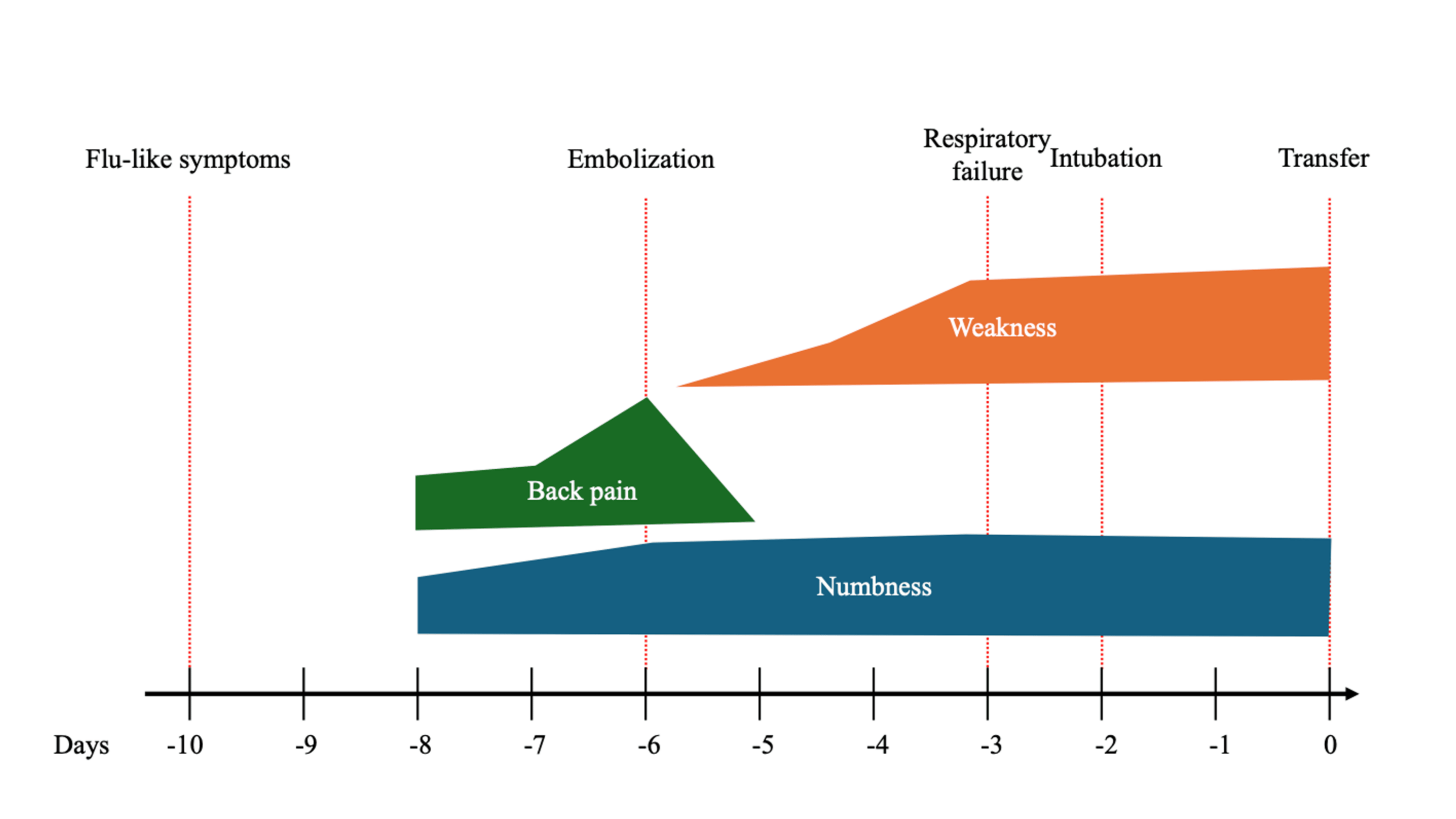
Summary of the clinical time course Two days after the onset of flu-like symptoms, the patient developed back pain and numbness. Two days later, the back pain worsened, and coil embolization of the pancreaticoduodenal artery aneurysm was performed. After embolization, the back pain resolved, whereas the numbness persisted and muscle weakness subsequently developed. Progressive weakness eventually resulted in respiratory failure requiring intubation and mechanical ventilation, prompting transfer to our hospital for further evaluation.

A 34-year-old woman was transferred to our hospital for evaluation of muscle weakness in all four limbs and respiratory failure following transcatheter arterial coil embolization for an impending rupture of a pancreaticoduodenal artery aneurysm. She had been well until 10 days before transfer, when she developed flu-like symptoms. Two days later, she noticed numbness involving all extremities and back pain. The numbness extended to her face the following day, prompting her to visit a nearby clinic. A head computed tomography (CT) scan and routine blood tests were performed; both were unremarkable, and an outpatient neurology consultation was scheduled. The next day, her back pain worsened substantially, and she was brought to a regional hospital by an ambulance. A contrast-enhanced abdominal CT scan revealed an impending rupture of a pancreaticoduodenal artery aneurysm associated with celiac artery stenosis (Figure 1). She underwent urgent transcatheter arterial coil embolization, after which her back pain improved. However, the numbness persisted, and generalized muscle weakness was noted after the procedure. These symptoms were initially attributed to the residual effects of the sedative agents used during the procedure. Three days later, she developed worsening respiratory failure with hypercapnia, requiring intubation and mechanical ventilation. She was subsequently transferred to our hospital for further evaluation and therapy. Figure 2 shows a summary of the clinical time course.

On admission, her body temperature was 38.0 ℃. She was intubated and mechanically ventilated in assist-control mode with an FiO_2_ of 0.40; oxygen saturation was 100%. Other vital signs were within normal limits. Physical examination was unremarkable.

On neurological examination, she was alert, with a Glasgow Coma Scale (GCS) score of E4VTM6. Cranial nerve examination was unremarkable except for symmetrical facial weakness. Muscle strength testing revealed severe weakness in all four limbs, with a Medical Research Council (MRC) [11] grade of 1/5. No atrophy or fasciculation was observed. Tendon reflexes were absent throughout. She reported dysesthesia involving all extremities.

**Figure 3:**
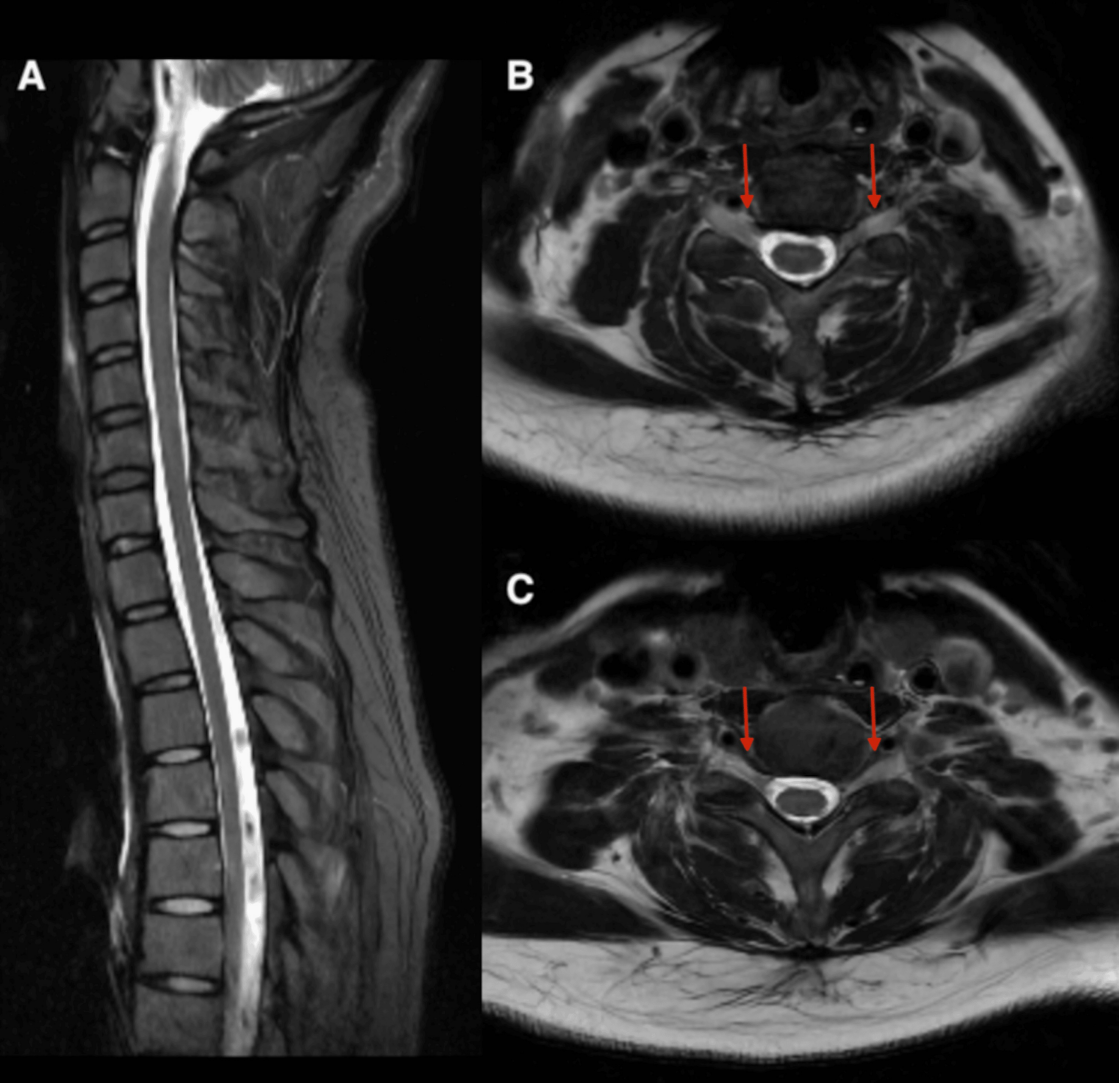
T2-weighted image of cervicothoracic spine MRI: (A) sagittal view, (B) axial view at the C5/6 level, and (C) axial view at the C6/7 level No structural abnormalities were observed on the sagittal view. (B) and (C) show bilateral enlargement of the nerve roots (red arrows).

**Figure 4:**
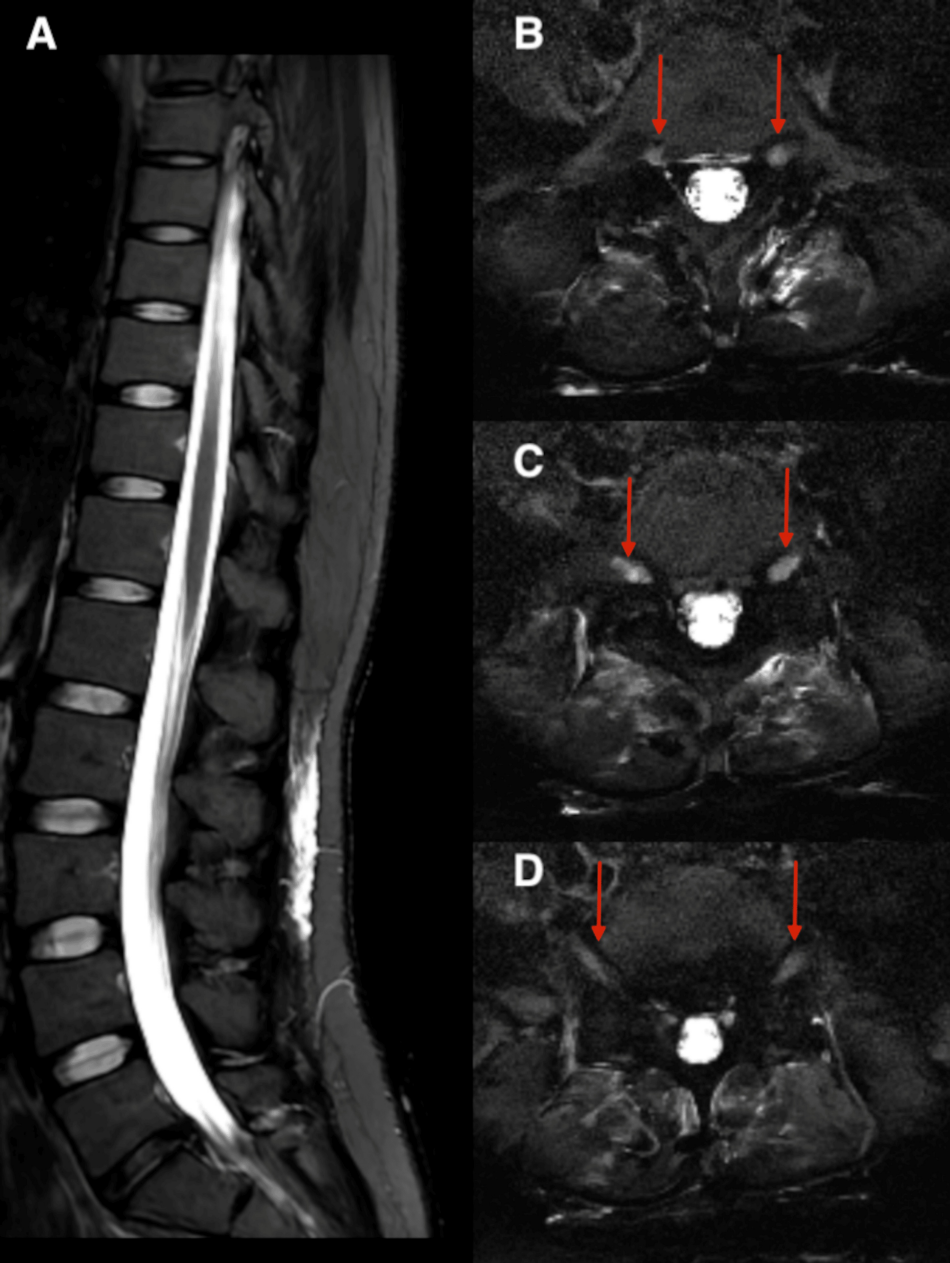
Short tau inversion recovery image of thoracolumbar spine MRI: (A) sagittal view and (B-D) serial axial view at the L5/S1 level No structural abnormalities were observed in the sagittal view. (B-D) The images show bilateral enlargement of the nerve roots (red arrows).

Laboratory tests were unremarkable except for an elevated white cell count of 11.9 (normal 3.6-8.9) × 10^9^/L, an elevated C-reactive protein level of 6.7 (normal <0.3) mg/dL, and a reduced vitamin B12 level of 158 (normal 180-914) pg/mL. Infectious workup was negative, including hepatitis B surface antigen, hepatitis C virus antibody, HIV-1/2 antibody, and both non-treponemal and treponemal tests for syphilis. Immunoglobulin levels were within normal limits. Tests for autoantibodies, including AChR antibody, antinuclear antibody, PR3-ANCA, MPO-ANCA, anti-SS-A antibody and anti-SS-B antibody were negative. Cerebrospinal fluid (CSF) analysis revealed a cell count of 11 (normal <5) cells/μL, a protein level of 230 (normal 10-40) mg/dL, and a glucose level of 83 mg/dL (serum glucose, 117 mg/dL). Brain magnetic resonance imaging (MRI) did not show any abnormalities. MRI of the spine showed bilateral enlargement of the lower cervical and lumbosacral nerve roots on T2-weighted (Figure 3) and short tau inversion recovery (STIR) images (Figure 4), respectively. Nerve conduction study and electromyography could not be performed because of her critical condition requiring intubation and mechanical ventilation.

Based on a rapid progression of symmetrical muscle weakness, absent tendon reflexes, enlarged nerve roots on spine MRI, and exclusion of other etiologies, a diagnosis of GBS was made. The markedly elevated CSF protein concentration supported the diagnosis, while the mild CSF pleocytosis was considered compatible with GBS [12]. Intravenous immunoglobulin therapy (IVIG) was subsequently administered at 400 mg/kg/day for five days.

Approximately 10 days after the initiation of IVIG, muscle strength in the distal upper extremities slightly improved. At that time, serum antiganglioside antibody testing performed on admission returned positive results for anti-GM1 antibodies, with a cutoff index (COI) of 1.40 (normal <0.7) on ELISA.

A second course of IVIG was administered because of the limited clinical response to the first course. Following the second course of IVIG, muscle strength gradually improved. Approximately 60 days from admission, she was successfully weaned from mechanical ventilation. By hospital day 80, her muscle strength had substantially improved to an MRC grade of 3/5 in the upper extremities and 4/5 in the lower extremities. She was able to walk with a walker and was transferred to a rehabilitation facility.

## Discussion

Guillain-Barré syndrome manifests as an acute immune-mediated polyradiculoneuropathy [1]. The typical clinical course is characterized by progressive, symmetrical muscle weakness with areflexia that reaches its plateau within four weeks [1] and is often preceded by an infectious illness [13]. It has a heterogeneous clinical course and prognosis. Approximately 30% of cases show respiratory muscle weakness requiring mechanical ventilation [14]. This case illustrates a typical clinical presentation in which flu-like symptoms preceded the onset of numbness, followed by progressive weakness involving all four limbs and the respiratory muscles, resulting in respiratory failure. Although the patient had a low vitamin B12 level, vitamin B12 deficiency was considered unlikely to account for the numbness, weakness, and areflexia because of their rapid progression.

GBS may not be a common disease for non-neurologists, but physicians in all specialties should be familiar with this condition. This is important because GBS cases presenting after surgery or invasive procedures have been reported [2,3]. In those situations, a high index of suspicion is necessary because the use of anesthetic and sedative agents makes neurological evaluation difficult [4], and timely treatment has been associated with better outcomes [5]. In our patient, persistent muscle weakness and numbness after embolization of an aneurysm, which were initially interpreted as the residual effects of sedative medications, eventually led to the diagnosis.

Celiac artery stenosis may reduce the blood flow to the bowel, causing symptoms such as postprandial abdominal pain and weight loss [6]. Celiac artery stenosis may also be associated with the formation of visceral arterial aneurysms [8]. Reduced flow is compensated by collateral circulation, helping maintain blood flow to the celiac artery territory. However, the resulting changes in hemodynamic stress may weaken the arterial walls and contribute to the formation of true aneurysms in the pancreaticoduodenal arcades and dorsal pancreatic artery [7]. Although our patient denied any previous episodes of typical symptoms, an impending rupture of a pancreaticoduodenal artery aneurysm secondary to celiac artery stenosis was diagnosed based on the radiographic findings. Given the resolution of back pain after coil embolization, the exacerbation of back pain was presumed to be associated with the aneurysm.

The relationship between the two distinct conditions remains unclear. One possible explanation is that autonomic dysfunction associated with GBS might theoretically affect vascular hemodynamics and contribute to the impending rupture of the aneurysm. In fact, Hijikata et al. reported a case of subarachnoid hemorrhage due to the rupture of a de novo dissecting vertebral artery aneurysm after developing GBS and suggested that the hypertension caused by GBS might contribute to the rupture [15]. However, there is no direct evidence to support this hypothesis. Although a causal relationship between the two disorders cannot be denied, the simultaneous onset of the back pain and numbness suggests that the coexistence in our patient was most likely coincidental.

Hickam’s dictum is one of the most famous diagnostic principles used by physicians [10]. It emphasizes that patients may have multiple simultaneous conditions. In line with this principle, although it is extremely rare, two life-threatening conditions may coexist coincidentally. A delay in the diagnosis may have serious consequences in those situations. In this case, the persistent muscle weakness, respiratory failure, and numbness after the treatment of the impending aneurysm rupture prompted reassessment of the underlying pathology and led to the diagnosis of GBS. Hence, clinicians should not exclude the possibility of coexisting diseases when a single diagnosis does not fully explain the clinical course.

## Conclusions

When GBS develops during the periprocedural period, neurological evaluation may be complicated by the effects of sedative medications. Because physicians in various specialties may encounter such cases, they should be able to recognize the clinical features of GBS to facilitate timely treatment. Furthermore, two life-threatening conditions may co-occur by chance. As delays in diagnosis and treatment may result in serious consequences, clinicians should consider this possibility when a single disease fails to explain the entire clinical course.
